# *In Vivo* Fluorescence Lifetime Imaging Monitors Binding of Specific Probes to Cancer Biomarkers

**DOI:** 10.1371/journal.pone.0031881

**Published:** 2012-02-23

**Authors:** Yasaman Ardeshirpour, Victor Chernomordik, Rafal Zielinski, Jacek Capala, Gary Griffiths, Olga Vasalatiy, Aleksandr V. Smirnov, Jay R. Knutson, Ilya Lyakhov, Samuel Achilefu, Amir Gandjbakhche, Moinuddin Hassan

**Affiliations:** 1 National Institute of Child Health and Human Development, National Institutes of Health, Bethesda, Maryland, United States of America; 2 National Cancer Institute, National Institutes of Health, Bethesda, Maryland, United States of America; 3 Department of Molecular Biology, The John Paul II Catholic University of Lublin, Lublin, Poland; 4 Imaging Probe Development Center, National Institutes of Health, Rockville, Maryland, United States of America; 5 National Heart, Lung and Blood Institute, National Institutes of Health, Bethesda, Maryland, United States of America; 6 Department of Radiology, Washington University, St. Louis, Missouri, United States of America; Enzo Life Sciences, Inc., United States of America

## Abstract

One of the most important factors in choosing a treatment strategy for cancer is characterization of biomarkers in cancer cells. Particularly, recent advances in Monoclonal Antibodies (MAB) as primary-specific drugs targeting tumor receptors show that their efficacy depends strongly on characterization of tumor biomarkers. Assessment of their status in individual patients would facilitate selection of an optimal treatment strategy, and the continuous monitoring of those biomarkers and their binding process to the therapy would provide a means for early evaluation of the efficacy of therapeutic intervention. In this study we have demonstrated for the first time in live animals that the fluorescence lifetime can be used to detect the binding of targeted optical probes to the extracellular receptors on tumor cells *in vivo*. The rationale was that fluorescence lifetime of a specific probe is sensitive to local environment and/or affinity to other molecules. We attached Near-InfraRed (NIR) fluorescent probes to Human Epidermal Growth Factor 2 (HER2/neu)-specific Affibody molecules and used our time-resolved optical system to compare the fluorescence lifetime of the optical probes that were bound and unbound to tumor cells in live mice. Our results show that the fluorescence lifetime changes in our model system delineate HER2 receptor bound from the unbound probe *in vivo*. Thus, this method is useful as a specific marker of the receptor binding process, which can open a new paradigm in the “image and treat” concept, especially for early evaluation of the efficacy of the therapy.

## Introduction

One of the most important factors in choosing a proper treatment strategy for cancer is characterization of biomarkers in cancer cells, and this can be rather challenging. Particularly, recent advances in Monoclonal AntiBodies (MAB) as primary-specific drugs, targeting tumor receptors, show that their efficacy depends strongly on overexpression of specific receptors.

Furthermore, one of the important factors that is frequently missing in cancer treatment (mainly using MAB) is continuous monitoring the response of the tumor receptors to the therapy, especially at the early stages, to quantify the binding process, which is critical for evaluation of drug efficacy.

Current standard *ex vivo* methods, e.g., ImmunoHistoChemistry (IHC), gene amplification demonstrated by Fluorescent *In Situ* Hybridization (FISH), and Enzyme-Linked ImmunoSorbent Assay (ELISA), are invasive and require biopsies from the patients. Inherently, biopsies have a risk of missing the malignant lesion and, during the therapeutic cycle, the number of times that the biopsy can be taken is limited [1]. Alternative methods currently under consideration are based on pharmacokinetics of the radionuclide probes in PET after injection into the blood circulation [2]. *In vivo* fluorescence imaging is an alternative non-invasive imaging technique, which can be used separately or in adjunction with other modalities for timely monitoring of the biomarker during the course of treatment. This method is simple and portable.

Recent advances in fluorescent probes, targeting specific disease biomarkers, have opened a new era in *in vivo* fluorescence imaging. They make it a promising tool for medical diagnostics [3]–[8]. Particularly, development of Near InfraRed (NIR) fluorescent probes has significantly improved the capability of *in vivo* fluorescence imaging, due to low autofluorescence background and deep penetration of the NIR light in the tissue [9].

Fluorescence imaging can be realized in the form of measuring the fluorescence intensity distributions and/or the fluorescence lifetime [10]–[18]. Fluorescence lifetime imaging is based on evaluation of the average time that electronically excited fluorophore stays in the excited state before its transition to a ground state, accompanied by photon emission. Fluorescence lifetime can be measured by time-domain or frequency domain techniques [19]–[22]. In this study, we used the more accurate former method and measured the exponential transient decay of the fluorescence intensity with time after considering the effect of the impulse response of the system. It has been shown that fluorescence lifetime is independent of the concentration of the fluorophores and the intensity of the excitation light. Fluorescence lifetime can remain constant even within fivefold fluctuation in the intensity of the excitation light [23]. On the other hand, it can be sensitive to local biochemical environment, e.g., temperature and pH or molecular interactions [24], [25]. This property makes the fluorescence lifetime imaging a promising candidate for detecting and monitoring specific cancer receptors in the diagnosis and treatment of diseases. Another important application of this technique is to investigate the effectiveness of early-phase treatment response by monitoring the binding of drug molecules to the tumor cells.

In this study, we targeted the Human Epidermal Growth Factor 2 (HER2/neu) receptor, which is one of the important biomarkers in many cancers, including breast and ovarian cancer [26]. Overexpression of this receptor is correlated with poor prognosis and resistance to specific chemotherapies [27]. To optimize the treatment procedure, it is important to assess the expression of the HER2 receptor in the diagnostic process and to monitor it *in vivo* over the course of treatment. To assess status of this receptor, we applied a HER2-specific Affibody conjugated to near infrared (NIR) fluorescent dye.

Though several recent studies have shown improved Signal/Noise ratio for *in vivo* fluorescence imaging by caging the fluorophore dyes using Polymersomes [28], nanotubes [29] or using nanoparticles [30] as an alternative for a single molecule fluorophores, Affibody-DyLight750 conjugate, investigated in the manuscript, seems to be a better suited probe for our goal, i.e., characterization of HER2 receptors overexpression in tumors *invivo.*

Affibody molecules [31]–[34] are very stable proteins. They are relatively small (8.3 kDa), about 20 times smaller than antibodies. Like any other small molecules, they can accumulate in the tumor through the leaky tumor vasculatures. Their major diagnostic advantage is that they can be made highly specific to particular cancer cell receptors, for example, HER2. After binding to these receptors, the unattached probes wash out and mainly bound fluorescent ligands contribute to the signal from the tumor area at later times. This significantly improves the contrast of the tumor, compared to the background.

In our previous study, we have shown that the dynamic changes in fluorescence intensity levels of HER2-specific Affibody proteins, conjugated with a fluorescent dye, can be used to quantify the HER2 expression in tumor cells [35]–[37]. Relatively fast pharmacokinetics of the Affibody-DyLight750 conjugate accelerates data collection and simplifies their analysis. The method, presented in the current manuscript, though less quantitative at the current stage comparing to pharmacokinetics analysis, allows one to assess the HER2 status of the tumor from just one time-resolved measurement. It does not require repeating measurements of the fluorescence from the tumor after the probe injection that imply much longer times of observation. Suggested approach can be used for a preliminary assessment of HER2 expression in tumor. It is complimentary to quantitative characterization of HER2 receptors, based on pharmacokinetics.

On the other hand, it has been shown in our earlier studies [35] that the HER2 Affibody binds to a different epitope of the HER2 receptor than the epitope, targeted by such widely used monoclonal antibodies as trastuzumab or pertuzumab. This enables monitoring of HER2 expression during the therapy without interference with the potential effect of these drugs [35].

In this study, we investigated in live animals whether binding the Affibody-conjugated fluorescent probe to the HER2 receptor can influence fluorescence lifetime of the probe. For this purpose, the fluorescence lifetime were studied in three different cases: HER2-specific Affibody (His6-Z_HER2_:GS-Cys) fluorescent probe in human tumor models with high HER2 expression, HER2-specific Affibody fluorescent probe in a human tumor model with no HER2 expression, and HER2-nonspecific Affibody (His_6_-Z_Taq_:GS-Cys) fluorescent probe in human tumor models with high HER2 expression, all in the mouse model *in vivo*. The results reveal significant differences in the fluorescence lifetimes between the tumor area and the contralateral site, when and only when, the binding of optical probe to the tumor receptors could occur. To the contrary, no change in fluorescence lifetime was observed in the cases where the optical probes have no affinity to the HER2 receptors.

## Materials and Methods

### Contrast Agent

In these experiments, two different Affibody® molecules (Affibody, Stockholm, Sweden) were used: HER2-specific Affibody (His6-Z_HER2_:GS-Cys) and HER2-nonspecific Affibody (His_6_-Z_Taq_:GS-Cys), both conjugated with the Dylight750 fluorescent probe (Thermo-Fisher-Scientific,Waltham, Massachusetts). Dylight750 is bright enough to be used for *in vivo* studies without any modification and can easily be conjugated to the HER2 specific/non-specific Affibodies. The conjugation ratio of Dylight750 to affibody protein is 1∶1.

**Figure 1:**
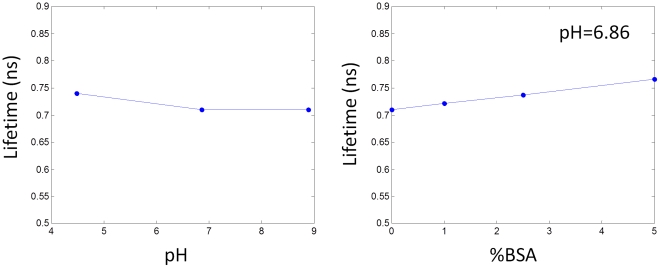
Effect of (A) pH and (B) BSA concentration on the lifetime of DyLight750 conjugated to HER2 specific Affibody probe.

We measured the lifetime of Affibody probe in chemical buffers (with a composition of citric acid, boric acid, and mono-sodium phosphate ranging from a pH of 4.5 to 9. No significant change in the fluorescence lifetime was observed over the whole range of pH (Fig. 1A). Sterilized saline solution was used for 3 times dilution of the Affibody probe before injection.

We also measured the fluorescence lifetime of Affibody probe in chemical buffer with pH of 6.86 by increasing its BSA concentration from 0% to 5% (Bovine Serum Albumin, Fraction V, Minimum 96%, Sigma Co.). The lifetime of the Affibody probe proved to be sensitive to the presence of BSA proteins (Fig. 1 B). We have not observed a decrease in the fluorescence lifetime relative to the Affibody probe, used for injection.

### Cell culture

For animal studies, three human tumor xenograft models expressing different levels of HER2 were used in this study. BT-474 and NCI-N87 are human tumor cell lines with a high level of HER2 (HER2/neu) expression: 243±24 ng/mg and 395±41 ng/mg protein, respectively, while MDA-MB-468 is a human tumor cell line with no HER2 expression. All three cell lines were obtained from American Type Culture Collection (ATCC; Manassas, VA). For in-vitro study, we have used either SK-BR-3 cell line with high level of HER2 expression or HER2-negative MDA-MB-468 human cancer cell line. SK-BR-3 has been chosen among HER2 positive cell lines because of its better attachment to the plate. The cells were grown in RPMI (BT-474, NCI-N87), DMEM-F12 (SK-BR-3, MDA-MB-468) culture media supplemented with 10% fetal bovine serum (FBS) and Pen/Strep (10,000 U penicillin, 10 mg streptomycin) at 37°C at 5% CO_2_ in a humidified incubator. Solution of 0.05% trypsin and 0.02% EDTA was used for cells detachment.

### *In vitro* study

HER2-positive SK-BR-3 and HER2 negative MDA-MB-468 cells were placed on 8-chambered confocal slides and incubated overnight. After 24 hours incubation, Affibody-DyLight488-conjugate was added to cells media at 0.5 µg/ml concentration. After additional 30 min incubation at 37°C, 2 µg/ml of Hoechst 33342 (Invitrogen, Carlsbad, CA) were added to the cells and incubated for another 1 hour for nuclei staining. Then cells were washed three times and imaged in PBS by Zeiss LSM 510 confocal microscope (further details of the experiment will be presented elsewhere [in preparation]). We have used marker similar to Affibody-DyLight750-conjugate (Affibody-DyLight488), because Zeiss LSM 510 confocal microscope does not operate at the near infrared wavelengths.

Additionally, to confirm the consistency of obtained results with Affibody-DyLight750-conjugate and evaluate the lifetime of the Affibody probe *in vitro*, FLIM microcopy of the cell cultures was performed by a specially adapted Olympus FV1000 inverted laser-scanning two-photon microscope [38] for imaging through non-descanned detection pathway in one-photon mode. Even though, these modifications reduced the microscope depth resolution significantly compared to confocal microscopy, it allowed to demonstrate clearly binding of HER2-Affibody-DyLight750-conjugate to the outer membrane of HER2 positive SK-BR-3 cells.

Briefly, for imaging, an Olympus Fluoview 1000 galvo-mirror laser scanning microscope equipped with a wide-band tunable “Mai Tai DeepSee” Ti-Sapphire laser (by Newport Spectra-Physics) was used. The laser was set to a one-photon excitation wavelength of 717 nm (to minimize the scattering by dichroic mirror). Substantial laser attenuation with a combination of orthogonal calcite polarizer and a metallic neutral density filter was used to avoid detector saturation and sample photobleaching. Sample was mounted on an inverted stage in an 8-well plate and illuminated with a long-pass 740–830 nm dichroic mirror (FF741-Di01 by Semrock) by means of a water-immersion 0.8 NA LUMPlanFl N 40× objective from Olympus. Two stacked Newport Oriel 51350 color glass 780 nm longpass filters were used in front of a red-sensitive Peltier-cooled fast-rise PMT module (Becker & Hickl model PMC-100-20) to eliminate scattering, second harmonic generation and cells autofluorescence. Since the detector was mounted on a non-descanned side port without any special filtering, effectively Z-resolution was decreased significantly in favor of collection efficiency. A fast-response silicon photodiode (Becker & Hickl model PHD-400-N-12) was used for laser synchronization. Photon counting events were discriminated, registered and assigned to pixel locations by Becker&Hickl SPC-830 PCI board. Data analysis was performed with SPCImage ver. 3.2 software by Becker&Hickl. Typical photon accumulation times varied from 60 to 300 s employing fast bidirectional scanning mode to further reduce photobleaching. Average count rate was kept well under 2 MHz to avoid pulses pileup.

The instrument response function, generated from crushed urea crystals in oil, was detected as second harmonic generation (laser tuned to 820 nm) through a proper set of dichroic/emission filters and measured as FWHM ∼190 ps (in our case was determined by the PMT transit time spread).

### Animal Preparation

For animal studies, the cultured cancer cells were implanted into the right forelimb of xenograft female nude mice. Five million cells were injected in 0.1 mL of 50% Matrigel into the right forelimb. The study was approved by National Institutes of Health Animal Care and Use Committee (Approval ID: ROB117). After the tumors grew to the size of 0.5–1 cm, mice were transferred to our imaging facilities. Before imaging, the mouse was anesthetized by isoflurane inhalation. The mouse was placed on a temperature-controlled stage and two sets of images from the tumor region and contralateral site were captured before injection. Then, 10 µg of the Affibody conjugated to fluorescent probe was injected through the tail vein. Images were captured continuously every 30 min for the first 5 hours. Tumor tissue was extracted from animals 24 hours post HER2-Affibody-DyLight750 injection, fixed in 10% neutral burred formalin (NBF) (Sigma-Aldrich, St. Louis, MO). Tumors were stained for HER2 expression (Herceptest, Daco) and Affibody distribution pattern.

It should be noted that Affibody-DyLight compounds do not induce toxic effects on breast cancer cells, as was demonstrated by *in vitro* experiments with cell cultures (experimental details will be presented elsewhere [in preparation]). To further validate the low toxicity of the probe *in vivo*, we kept several mice with the injected doses as high as 0.5 mg/kg alive up to 1 month after injection of the Affibody-DyLight750 compound without observing any toxicity effects in the mouse.

### *In-vivo* Lifetime Imaging System

The *in-vivo* lifetime imaging system consisted of a tunable pulse laser with a pulse width of 100 fs and repetition rate of 80 MHz. The laser was tuned to an excitation wavelength of 750 nm. The light pulses scanned the target (tumor or contralateral site) of an animal in a raster pattern through a scanning head with source and detector fibers in 2 mm distance. The animal was placed within a dark chamber on a temperature-controlled scanning stage. The reflected fluorescence signal was filtered by a 780 nm long-pass emission filter. The detected photons were captured by a photomultiplier tube (PMT) and photons were counted by a time-correlated single-photon counter (TCSPC). Initialization, scanning, and acquisition were controlled by the Labview software. The details of the imaging system can be found in [15]. The fluorescence lifetime was estimated from a curve fitting of the data to a single exponential decay function, optimized by iterative reconvolution of single decaying exponential with the impulse response function of the system. We truncated the tail of the time-resolved intensity data due to its more susceptibility to photon transport path variations. In our preclinical studies, since the tumor is located in mouse forelimb subcutaneously, the depth of the tumor does not have a significant effect on the lifetime, however, for applications dealing with the deeply embedded tumors, the effect of the photon diffusion on the observed time-resolved fluorescence intensities should be taken into account.

## Results

### *In vivo* Study

To investigate the effect of binding the HER2-specific Affibody fluorescent probe to the HER2 receptor-positive tumor on fluorescence lifetime, three different cases were considered. In the first case, the HER2-specific Affibody (His6-Z_HER2_:GS-Cys) optical probe was injected in mice with high HER2-expressing human tumor carcinomas. In the second case, a HER2-nonspecific Affibody (His_6_-Z_Taq_:GS-Cys) fluorescent probe was injected in mice with the same HER2-expressing human tumor carcinomas as in the first case, and in the third case, the HER2 specific Affibody (His6-Z_HER2_:GS-Cys) fluorescent probe was injected in mice with no HER2-expressing tumor.

**Figure 2:**
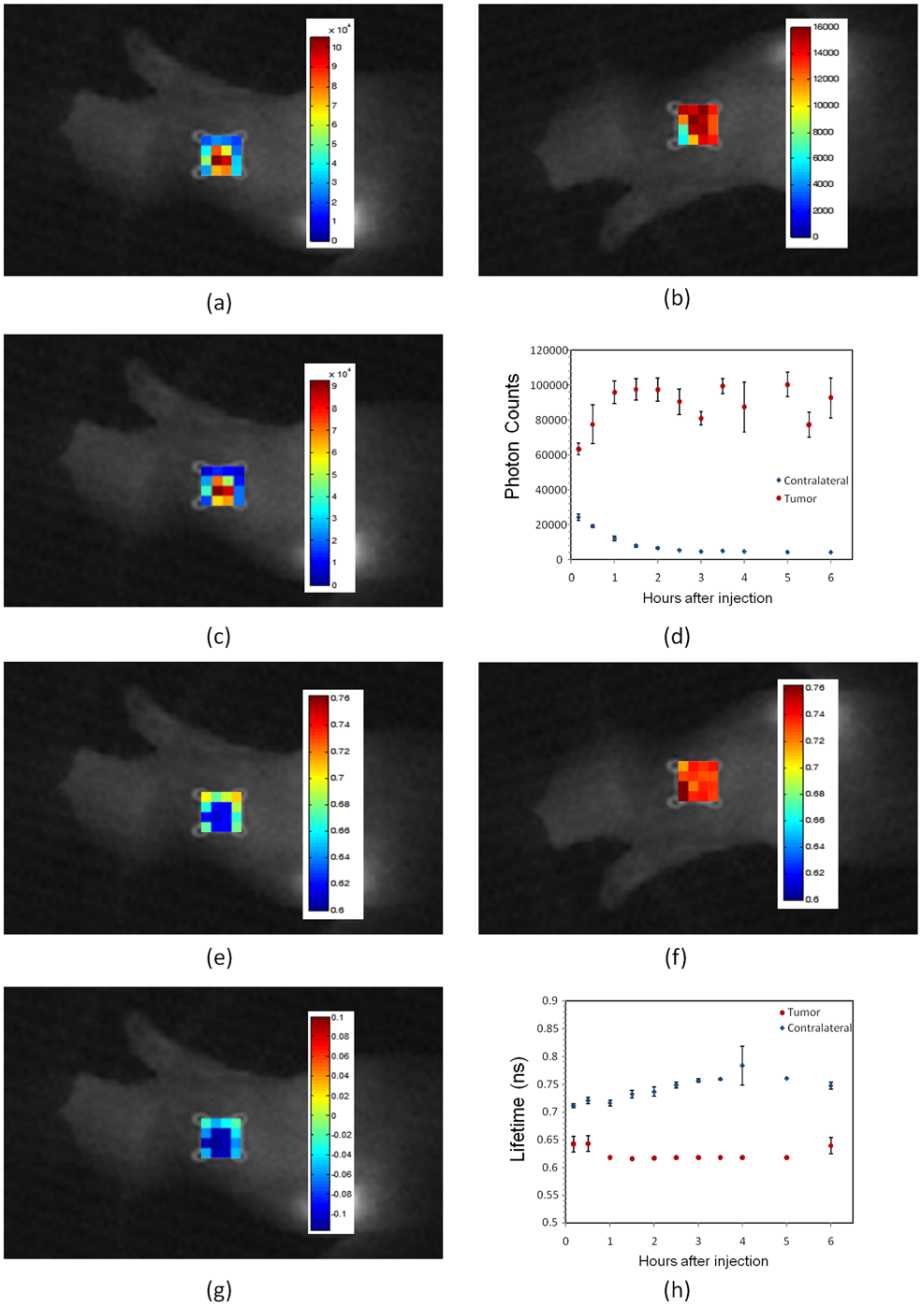
*In vivo* fluorescence imaging of xenograft mouse with high HER2 expressing human tumor model (BT-474) after injection of HER2-specific Affibody® (His6-Z_HER2_:GS-Cys) conjugated to Dylight750. (**A**) Fluorescence intensity map at the tumor region. (**B**)Fluorescence intensity map at the contralateral site (**C**) The difference of fluorescence intensity at the tumor region and the contralateral site, mapped on the tumor region. (**D**) Pharmacokinetics of the fluorescence intensity at the tumor region and contralateral site after the injection over time. The fluorescent intensity was averaged over 16 pixels at the contralateral site. The data in Figs. (**D**) and (**H**) are the average data of four mice. Markers show the average and bars show the standard deviation. (**E**) Fluorescence lifetime map at the tumor region. (**F**) Fluorescence lifetime map at the contralateral site. (**G**) The difference of fluorescence lifetime at the tumor and the contralateral site mapped on the tumor region. (**E**)Pharmacokinetics of the fluorescent lifetime at the tumor region and contralateral site after injection over time. All lifetime and fluorescence intensity maps in figures 2,4–7 are from measurements at 1 hour after the injection of Affibody probe. In all measurements, the photons were counted over two seconds integration time, *t_0_*. To exclude saturation effect of the 16 bit camera, in the brightest pixels, where the corresponding limit of 65536 counts was reached before time *t_0_*, we have renormalized the data by multiplying photon counts, measured for lower integration time *t*, by factor *t_0_/t.*

In the first experiment, HER2-specific Affibody was injected into four mice with a high level (+3) HER2-expressing human tumor carcinoma, BT-474. Fig. 2 shows the results of *in vivo* measurements of fluorescence intensity and lifetime at the tumor area and the contralateral site. Fig. 2C shows the difference between the fluorescence intensity at the tumor (Fig. 2A) and the contralateral sites (Fig. 2B) 1 hour after injection, mapped on the tumor area. Fig. 2D shows the dynamics of the maximum fluorescence intensity at the tumor region and contralateral site for 6 hours after the injection. The pixel with maximum intensity was used to characterize the tumor. The fluorescence mapping at the contralateral site was averaged over 16 pixels since there was no specific point to target at the contralateral site. Averaging over 16 pixels has been used for noise reduction. To demonstrate the variations of the lifetime and fluorescence intensity throughout the scanned area we presented maps of fluorescence intensity and lifetime at the contralateral and tumor regions. The data shown in the Fig. 2D and Fig. 2H are the average values of the measurements over 4 mice (Markers show the average and bars show the standard deviation). Fig. 2G shows the difference between the fluorescence lifetime at the tumor (Fig. 2E) and contralateral sites (Fig. 2F) 1 hour after injection, mapped on the tumor region. The fluorescence lifetime in the tumor area and contralateral site for over 6 hours after injection is shown in Fig. 2H. In Figs. 2H, the data, corresponding to the pixel with maximum intensity were used for lifetime calculations since it had the highest signal to noise ratio.

In all experiments, the data were captured from the tumor and contralateral sites at the same time range as the first experiment. For the fluorescence intensity and lifetime maps, we chose the measurement data at 1 hour after injection, corresponding to a case, when considerable amount of fluorescent dyes were available in both contralateral and tumor sites.

**Figure 3:**
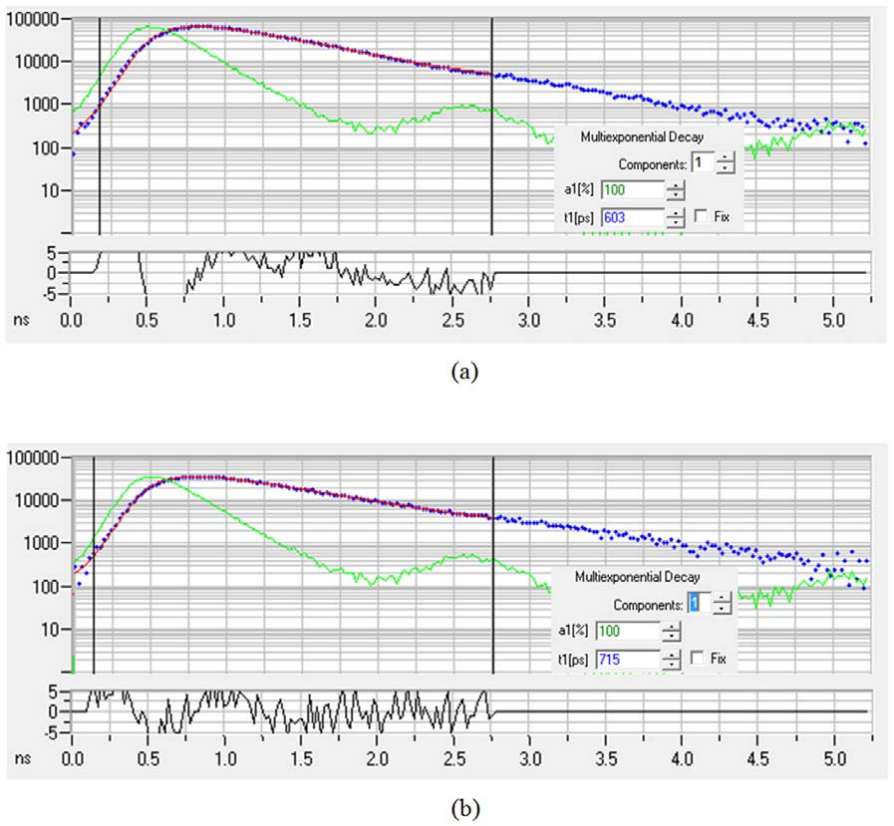
An example of fitting results obtained by SPCImage software, (ver. 3.2, Becker & Hickl GmbH) for measurements at the tumor (A) and contralateral (B) sites, 1 hour after injection of HER2-specific Affibody conjugated to Dylight750 in a mouse with high HER2 expressing human tumor model (BT-474). X axis is the amplitude and y axis is the measurement time (ns). Blue and green graphs show the measurement data and impulse response function of the system, respectively. The fitting was based on single exponential decay model. Parameter a1 shows the relative of amplitude in single exponential decay model and t1 is the lifetime (in picoseconds). The small graph at the bottom is the fitting error and the red line shows the fitted curve.

As an example, the fitting curves obtained by SPCImage software, (ver. 3.2, Becker & Hickl GmbH) are shown in Fig. 3 for measurements both at the tumor (Fig. 3A) and contralateral (Fig. 3B) sites. These data are measurements, performed 1 hour after injection the HER2-specific Affibody, conjugated to DyLight750, in mouse with human tumor carcinoma xenograft (BT-474). This cell line is known to have high expression of HER2. Blue and green graphs show the measurement data and the impulse response function of the system, respectively. The fitting was based on single exponential decay model. Parameter a1 shows the relative of amplitude of the signal used in single exponential decay model and t1 is the lifetime (in picoseconds). The small graph at the bottom is the fitting error and the red line presents the fitted curve.

**Figure 4:**
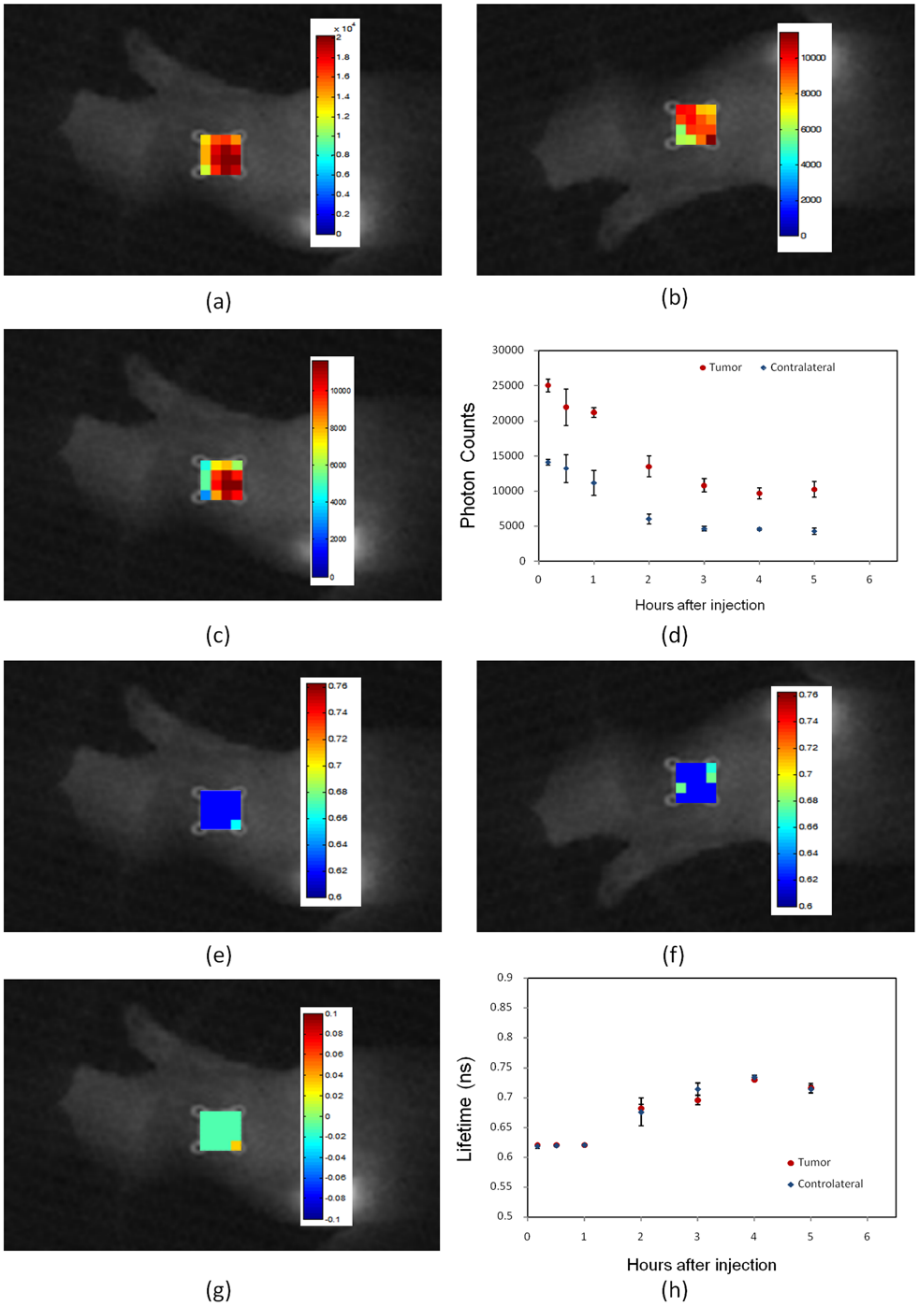
*In vivo* fluorescence imaging of xenograft mouse with high HER2 expressing human tumor model (BT-474) after injection of HER2-nonspecific Affibody® (His6-Z_Taq_:GS-Cys) conjugated to Dylight750. (**A**) Fluorescence intensity map at the tumor region. (**B**)Fluorescence intensity map at the contralateral site (**C**) The difference of fluorescence intensity at the tumor region and the contralateral site, mapped on the tumor region. (**D**) Pharmacokinetics of the fluorescence intensity at the tumor region and contralateral site after the injection over time. The data in Figs. (**D**) and (**H**) are the average data of three mice. Markers show the average and bars show the standard deviation. (**E**rpar; Fluorescence lifetime map at the tumor region. (**F**) Fluorescence lifetime map at the contralateral site. (**G**) The difference of fluorescent lifetime at the tumor region and the contralateral site mapped on the tumor region. (**E**) Pharmacokinetics of the fluorescent lifetime at the tumor region and contralateral site after injection over time.

In the second experiment, we have used three mice with the same HER2 positive carcinoma (BT-474). Contrary to the previous experiment, an HER2-nonspecific Affibody (His_6_-Z_Taq_:GS-Cys) optical probe was used as a contrast agent. The results are shown in Fig. 4. Due to non-specificity of Z_Taq_ affibody to HER2 receptors, no probe binding/accumulation in HER2 positive tumors was observed in this experiment.

**Figure 5 pone-0031881-g005:**
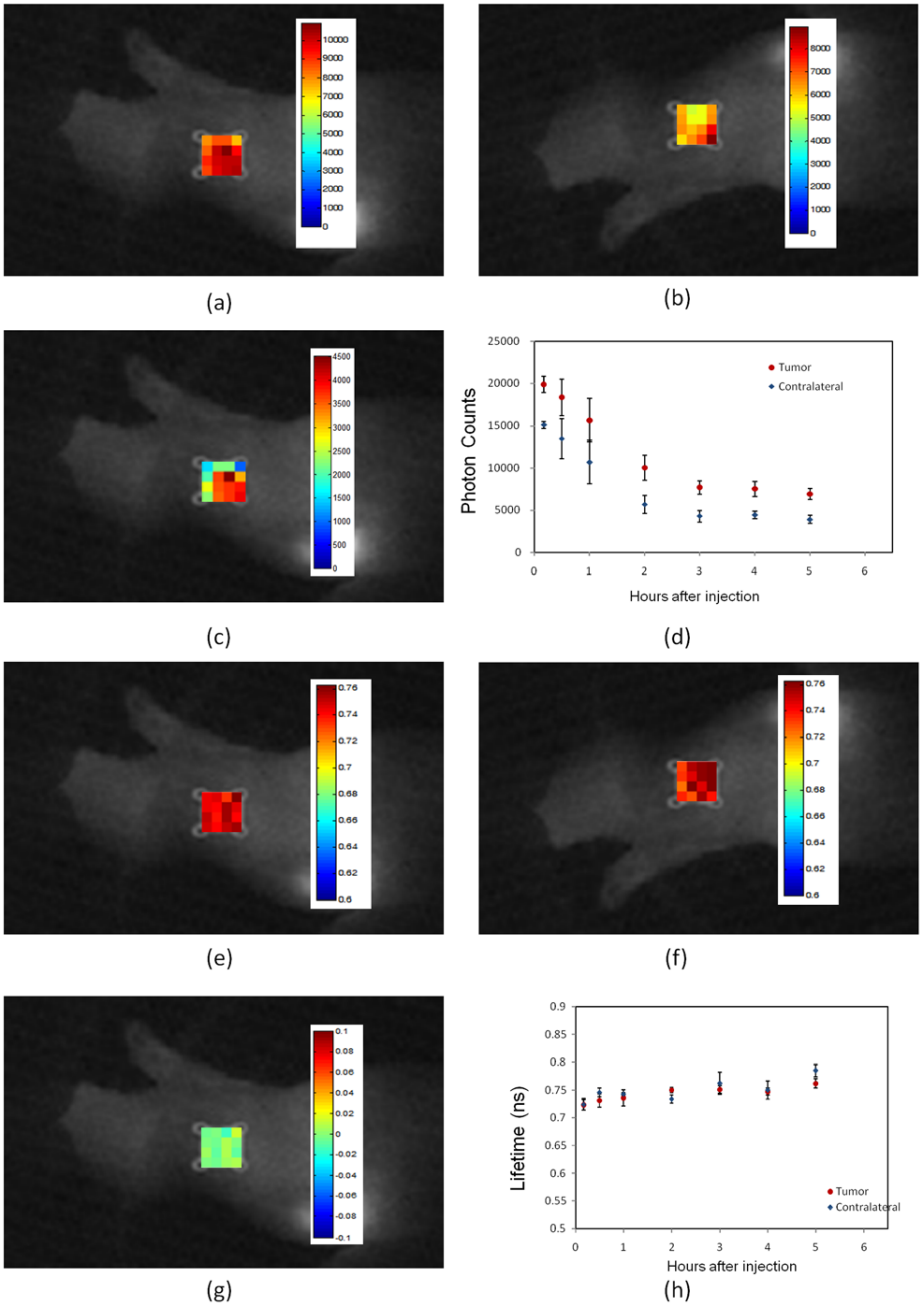
*In vivo* fluorescence imaging of xenograft mouse with no HER2 expressing human tumor model (MDA-MB-468) after injection of the HER2-specific Affibody® (His6-Z_HER2_:GS-Cys) conjugated to Dylight750. (**A**) Fluorescence intensity map at the tumor region. (**B**) Fluorescence intensity map at the contralateral site. (**C**) The difference of fluorescence intensity at the tumor region and the contralateral site, mapped on the tumor region. (**D**) Pharmacokinetics of the fluorescence intensity at the tumor region and contralateral site after the injection over time. The data in Figs. (**D**) and (**H**) are the average data of three mice. Markers show the average and bars show the standard deviation. (**E**)Fluorescence lifetime map at the tumor region. (**F**) Fluorescence lifetime map at the contralateral site. (**G**) The difference of fluorescent lifetime at the tumor and the contralateral site mapped on the tumor region. (**E**) Pharmacokinetics of the fluorescent lifetime at the tumor region and contralateral site after injection over time.

For the third experiment, three mice with the HER2-negative model (MDA-MB-468) were used for the study. Again the HER2-specific Affibody (His6-Z_HER2_:GS-Cys) optical probe was used as a fluorescent contrast agent. In this specific human tumor model, due to the lack of the HER2 receptors in the tumor area, HER2 Affibody did not bind to the tumor cells. The intensity at both tumor and contralateral site decreased significantly in 2–3 hours after injection (Fig. 5D). The fluorescence lifetime was also observed to be the same at both tumor and contralateral sites (Fig. 5H).

**Figure 6 pone-0031881-g006:**
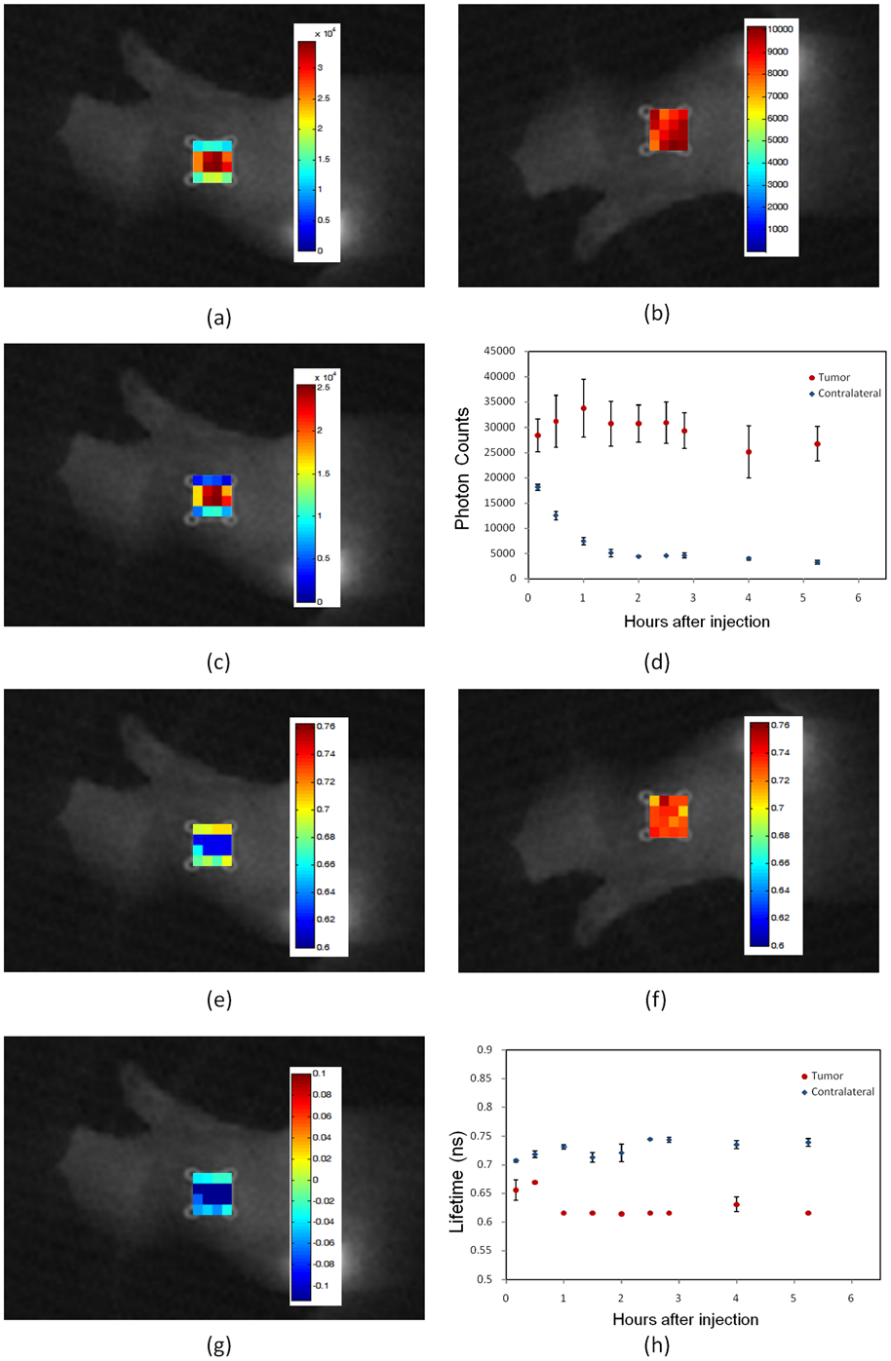
*In vivo* fluorescence imaging of xenograft mouse with high HER2-expressing human tumor model (NCI-N87) after injection of the HER2 specific Affibody® (His6-Z_HER2_:GS-Cys) conjugated to Dylight750. (**A**) Fluorescence intensity map at the tumor region. (**B**)Fluorescence intensity map at the contralateral site. (**C**) The difference of fluorescence intensity at the tumor region and the contralateral site, mapped on the tumor region. (**D**) Pharmacokinetics of the fluorescence intensity at the tumor region and contralateral site after the injection over time. The data in Figs. (**D**) and (**H**) are the average data of three mice. Markers show the average and bars show the standard deviation. (**E**)Fluorescence lifetime map at the tumor region. (**F**) Fluorescence lifetime map at the contralateral site. (**G**) The difference of fluorescent lifetime at the tumor and the contralateral site mapped on the tumor region. (**E**) Pharmacokinetics of the fluorescent lifetime at the tumor region and contralateral site after injection over time.

**Figure 7:**
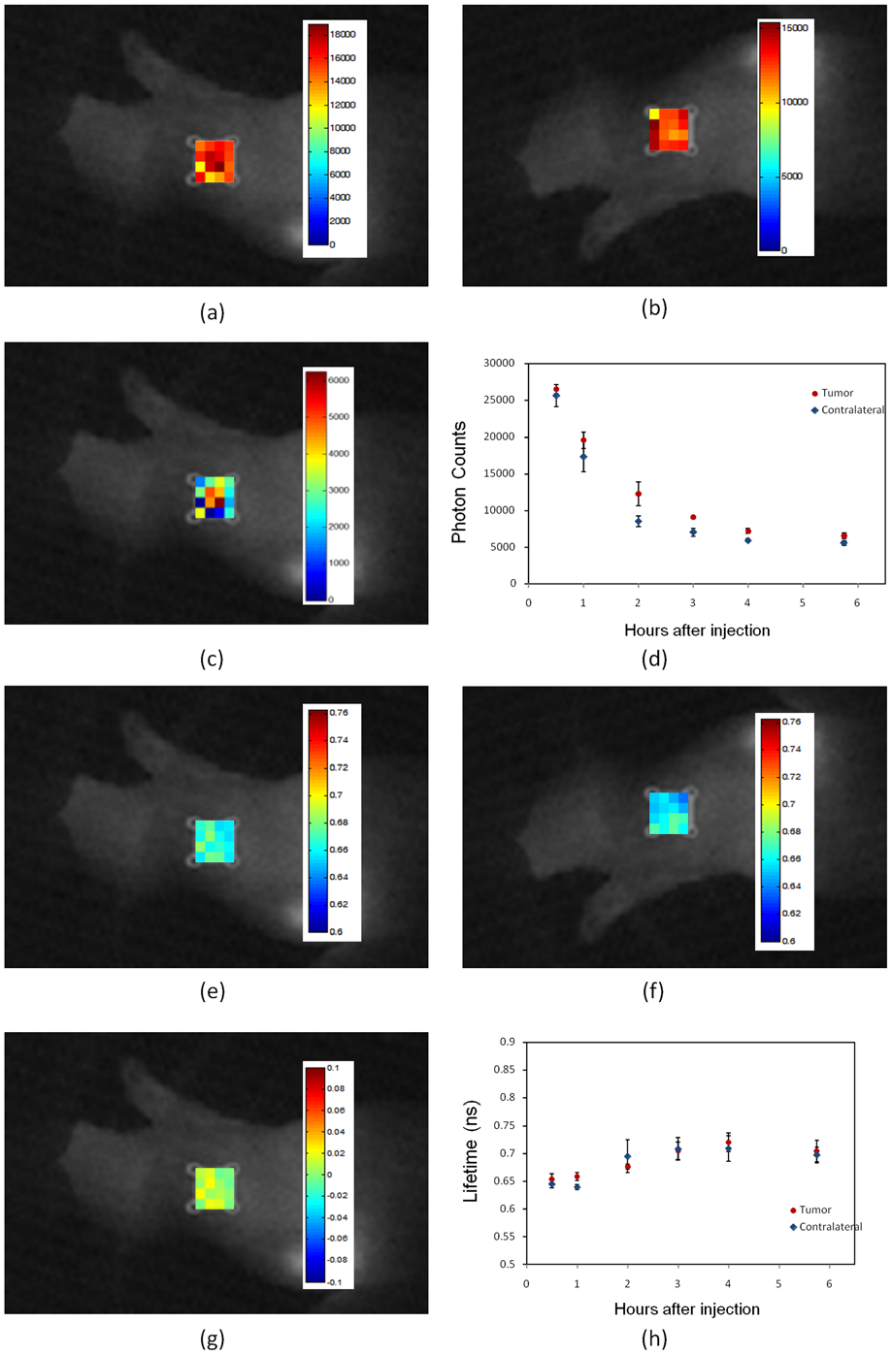
*In vivo* fluorescence imaging of xenograft mouse with high HER2 expressing human tumor model (NCI-N87) after injection of the HER2-nonspecific Affibody® (His6-Z_Taq_:GS-Cys) conjugated to Dylight750. (**A**) Fluorescence intensity map at the tumor region. (**B**)Fluorescence intensity map at the contralateral site (**C**) The difference of fluorescence intensity at the tumor region and the contralateral site, mapped on the tumor region. (**D**) Pharmacokinetics of the fluorescence intensity at the tumor region and contralateral site after the injection over time. The data in Figs. (**D**) and (**H**) are the average data of three mice. Markers show the average and bars show the standard deviation. (**E**)Fluorescence lifetime map at the tumor region. (**F**) Fluorescence lifetime map at the contralateral site. (**G**) The difference of fluorescent lifetime at the tumor and the contralateral site mapped on the tumor region. (**E**) Pharmacokinetics of the fluorescent lifetime at the tumor region and contralateral site after injection over time.

The same tests were repeated for the NCI-N87 tumor model. NCI-N87 cells are also known to have a high level of HER2 expression [26]. The fluorescence intensity and lifetime were measured *in vivo* after injection of fluorescent probe, based either on HER2-specific Affibody (His6-Z_HER2_:GS-Cys) or HER2-nonspecific Affibody (His6-Z_Taq_:GS-Cys). The results are shown in Fig. 6 and Fig. 7, respectively.

Before injection, the fluorescence lifetime of Dylight750, conjugated to HER2-specific Affibody (His6-Z_HER2_:GS-Cys), was measured as 0.71 ns. However, the lifetime of Dylight750 conjugated to HER2-nonspecific Affibody (His6-Z_Taq_:GS-Cys) was measured as 0.62 ns. This lifetime difference between HER2-specific and non-specific were consistence in our in-vivo measurements at contralateral site.

**Figure 8:**
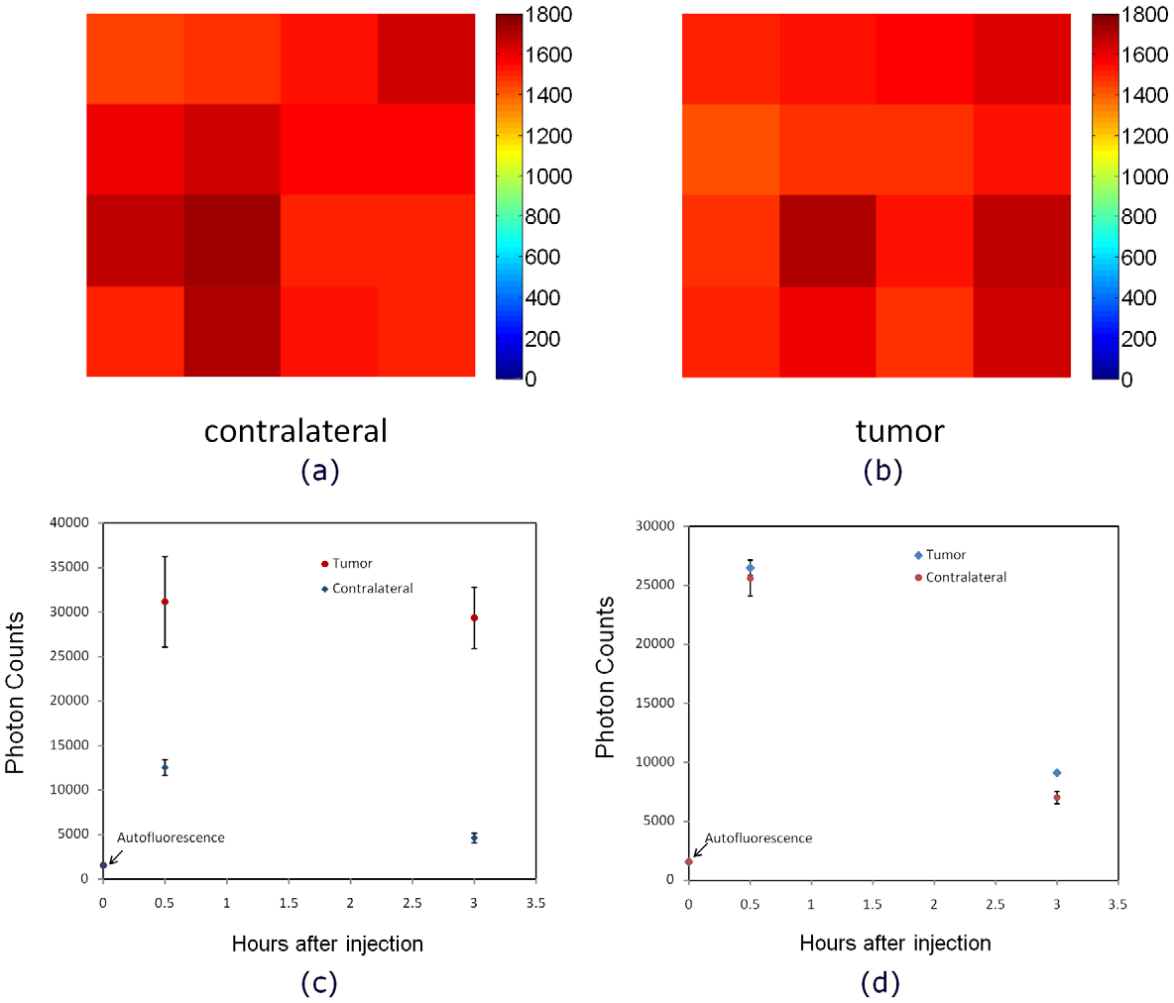
Comparison of autofluorescence intensity at the tumor and contralateral sites. The autofluorescence intensity map at the (**A**) tumor and (**B**) contralateral sites before injection. Comparison of the autofluorescence intensity before injection and the fluorescence signal after injection of (**C**) HER2 specific Affibody-Dylight750 and (**D**) HER2 non-specific Affibody-Dylight750 in 3 mice with HER2 positive tumor (NCI-N87 tumor carcinoma) at the tumor and contralateral sites .

To evaluate potential contribution of the autofluorescence into the measured fluorescence intensities, we have measured signal before the probe injection both at the tumor and contralateral sites. Figs. 8A–8B show intensity map of the 16 pixels at the tumor region and contralateral site before injection of the Affibody probe. A comparison between the autofluorescence (before injection) and the fluorescence signal after injection of (A) HER2 specific Affibody-Dylight750 (B) HER2 non-specific Affibody-Dylight750 in mice with NCI-N87 tumor xenografts is presented in Figs. 8C–8D for the tumor and the contralateral sites, respectively. These measurements confirm that the autofluorescence level is very low relative to the probe fluorescence signal, illustrating one of the major advantages of imaging in NIR region. In prescans (before probe injection), we were not able to distinguish the tumor region from the contralateral site (since the autofluorescence was lower than the noise floor of our system, lifetime estimates prove to be impossible in this case).

**Figure 9:**
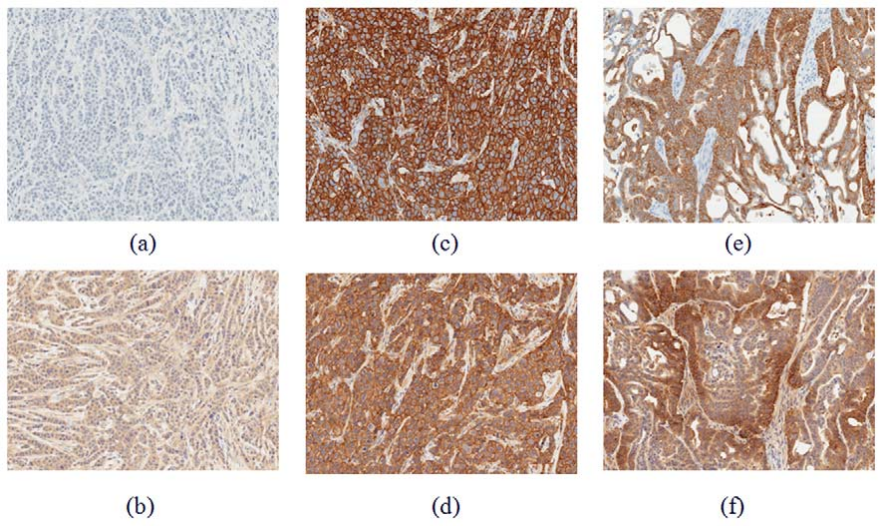
Histological (IHC) images of tumor tissues for tumor with no HER2 expression (A–B) MDA-MB-468, and highly expressed HER2 tumor (C–D) BT-474 and (E–F) NCI-N87. Tumor tissue was extracted from animals 24 hours post HER2-Affibody-DyLight750 injection, fixed in 10%NBF and analyzed by IHC for detection of HER2 status (**A,C and E**) and Affibody presence (**B,D and F**).

The histological images of extracted tumor tissues 24 hours after injection for tumor with no HER2 expression (9A–9B) MDA-MB-468, and tumors with high HER2 expression (9C–9D) BT-474 and (9E–9F) NCI-N87 are provided in Fig. 9.

To be more quantitative in the assessment of HER2 expression in the tumors we have also performed ELISA assays of the *ex vivo* tumor tissue, extracted 24 hours after initial *in vivo* fluorescence imaging. According to ELISA readings, the HER2 expression in HER2-negative tumor (MDA-MB-468) was negligible (0.92±0.17 ng/mg protein). To the contrary, in HER2-positive (3+) tumors BT-474, and NCI-N87, the measured HER2 expression was much higher, i.e., 243±24 ng/mg and 395±41 ng/mg, respectively.

### *In vitro* studies

**Figure 10:**
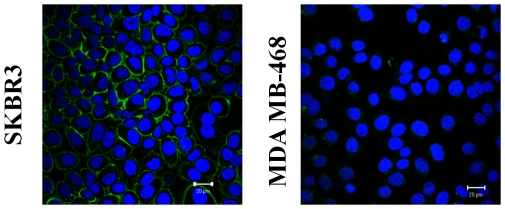
Confocal microscopy of HER2 positive (A) and negative (B) cells exposed to HER2-Affibody-DyLight488. Blue color shows the cell nuclei labeled with Hoechst 33342 and green color shows the HER2-Affibody-DyLight 488.

To understand the mechanism involved in the change in lifetime, we also tested the affinity and specificity of our probe to HER2 receptors *in vitro*, using cell cultures. It was shown by confocal microscopy that cancer cells, expressing high level of HER2 receptors, have evident membrane accumulation of the fluorescence dye after exposure to HER2-Affibody-Dylight488-conjugate. Neither membrane retention nor intracellular uptake of the probe was observed for HER2-negative MDA-MB-468 cells even after 24-hours of continuous exposure to HER2 targeting probe (Fig. 10).

**Figure 11:**
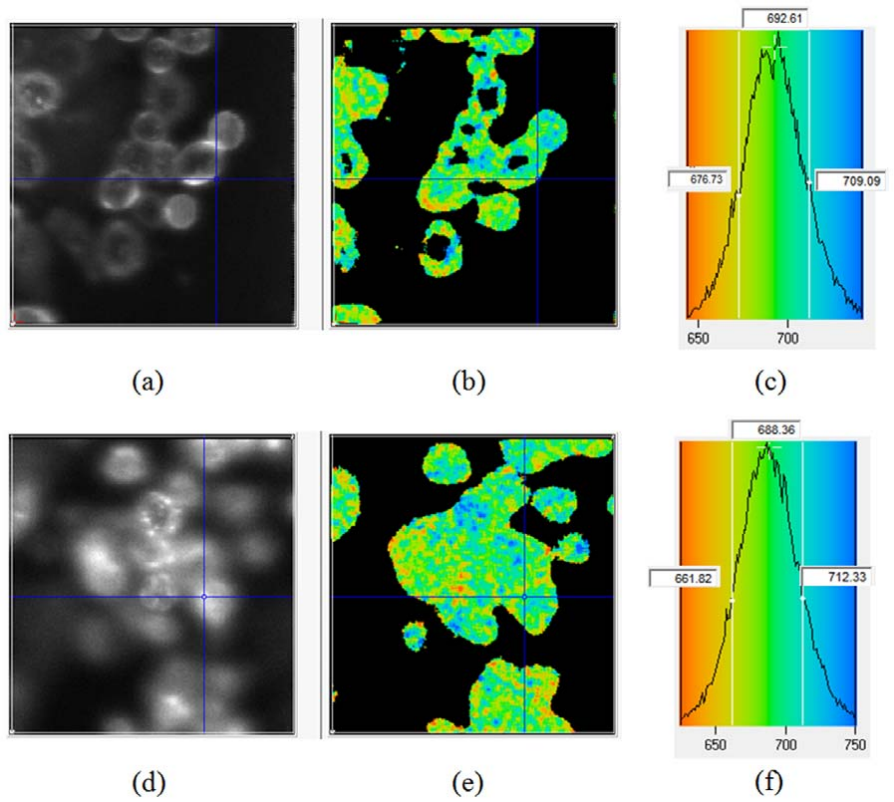
*In-vitro* image of HER2 positive cancer cells (SKBR3) exposed to HER2-Affibody-Dylight750 in (A–C) PBS and (D–F) cell culture with 10% FBS. Figures (**A**) and (**D**) show the intensity and (**B**) and (**E**) show the lifetime image. The histogram of the lifetime distribution has been shown in figures (**C**) and (**F**) for PBS and 10% FBS in cell culture media, respectively.

For *in vitro* imaging with modified FLIM microscope, we have also observed the binding of the HER2-Affibody-Dylight750 probes to the HER2 positive (3+) SK-BR-3 cells' outer membranes (Fig. 11D).

To elucidate the mechanism of lifetime changes due to Affibody binding to HER2 receptors, we imaged the cells within PBS and cell culture media with 10% FBS (the cells did not survive in 5% BSA solved in PBS). The data processing was performed with SPCimage software.

Figures 11A–11C and 11D–11F present the results on SK-BR-3 cell cultures in PBS and in cell culture media, containing 10% of FBS, respectively. Fluorescence intensity maps for both cases are shown in Figures 11A and 11D, while Figure 11B and 11E present corresponding fluorescence lifetime maps. The histograms in Figs. 11C and Fig. 11F illustrate observed distributions of the fluorescence lifetime for HER2-Affibody probe molecules, bound to SK-BR-3 cancer cells, for two considered types of cell media. The results reveal similar peak values and distribution for both cases.

The lifetimes of the dye in PBS, obtained with our FLIM system prove to be practically equal (712 ps). They are very close to what we have obtained for HER2 specific Affibody probe with our *in vivo* system before injection (∼710 ps).

## Discussion

This paper is focused on the effects of probe binding to cell receptors in *in vivo* system (mouse model of human cancer). We have studied three different scenarios, HER2 specific Affibody in tumor with no HER2 expression, HER2 specific Affibody in tumor with high HER2 expression and HER2 non-specific Affibody in tumor with high HER2 expression.

In human tumor models with high HER2 expression (Figs. 2 and 6), we observed a very high degree of accumulation of HER2-specific fluorescent dyes in the tumor volume for more than 24 hours after injection. In the same animals, the fluorescent probe washed out very fast from the contralateral site and the fluorescence intensity decreased exponentially with the decay time ∼1 hour. The fluorescence lifetime of the Affibody-Dylight750 conjugate at the contralateral site is close to that of before injection. To the contrary, the measured fluorescence lifetime was decreased significantly (∼100 ps) in the tumor area presumably because of binding to the tumor HER2 receptors.

In the second case (Figs. 4 and 7), His_6_-Z_Taq_:GS-Cys could not bind to HER2 receptors; therefore, the fluorophore probes were washed out from tumor as well, and the intensity was decreased in both tumor and contralateral regions after 2–3 hours. The results also show negligible difference between the fluorescence lifetime at the tumor and contralateral regions.

In the tumor model with no HER2 receptor expression (Fig. 5), the HER2-specific Affibody could not bind to the tumor cells. Therefore, the intensity at both tumor and contralateral sites decreased significantly in 2–3 hours after injection due to washout (Fig. 5C). The fluorescence lifetime was also observed to be the same at both the tumor and contralateral sites.

Statistical analysis substantiated these conclusions about qualitative differences between binding and no binding cases (see below).

Our preliminary *in vitro* experiments showed that it is not easy to reproduce the lifetime “binding” effect in cell cultures, probably because it is not simply binding to HER2 receptors per se that results in smaller lifetimes, but a combination of several processes, for example, involving the intermediate binding of the probe to blood albumin in circulation [39] with subsequent dissociation of specific probe inside the tumor from blood albumin through binding to HER2 receptors. For this reason, we believe that failure to reproduce “binding” effect *in vitro* up to now, have not put in doubt our *in vivo* findings; *in vivo* experiments were done with several independent controls: tumor region versus contralateral site, HER2-positive versus HER2-negative tumors, HER2-specific versus HER2 non-specific Affibodies. All *in vivo* results were very consistent. Our future *in vitro* studies will be focused on discovering the reasons for observed discrepancy between the live mouse and cell culture fluorescence data. Adequate mimicking of the cancer tumor microenvironment *in vitro* is very complicated and likely would require much more sophisticated setup.

The main difference between the three in-vivo study cases was the binding of HER2 Affibody to the HER2 receptors in the tumor. The other factors, like penetration of the fluorescence dye in the tumor area due to the leakiness of the tumor, the pH difference in the tumor and blood protein concentration should be the same in the case of same-type HER2-positive tumors, assessed with specific or non-specific Affibodies. On the other hand, for different tumor types, one could expect some differences in tumors' vascularity/leakiness, pH, blood protein etc. Thus, observed close similarity between the fluorescence lifetimes from tumor areas in the cases of HER2 positive tumors, imaged with non-specific probe, and HER2 negative tumors, imaged with similar, but HER2 specific probes (see Figs. 4, 7 and 6), further emphasizes the importance of the probe binding to HER2 receptors for fluorescence lifetime to decrease, indicating that all other mentioned above factors play relatively minor role. It should be also noted that in cases, not involving specific HER2 binding, fluorescence lifetime behaves similarly at the tumor and contralateral sites. We have shown that even at early times after injection, the fluorescence lifetime at the tumor is shorter in all cases, when we expect Affibody binding to the HER2 receptors, relative to other two control cases, or the contralateral site. Thus, our analysis of the observed differences between three studied cases indicates the major role of the binding of Affibodies to HER2 receptors. We have observed that wherever HER2 binding occurs (HER2 positive tumor + HER2 specific probe) fluorescence lifetimes at the tumor, 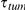, are shorter by more than 10%, comparing to that of the contralateral site, 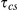. In addition, the temporal behavior of 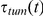 differs qualitatively from 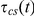, i.e., observed lifetimes at the tumor are practically constant 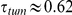 ns up to time *t*∼5 hours post injection after a small initial drop (∼0.02 ns) during initial *t*∼0.5 hours, while 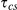 slowly increases from ∼0.7 ns to ∼0.75 ns after probe injection (see part H in Figs. 2,4–7).

In the opposite case (control experiments), when no HER2 binding of the probe can happen, either because the tumors are HER2 negative, or the tumors are HER2 positive (of the same types, as analyzed before), but the probe is HER2 non-specific (i.e., Affibody probe with no affinity to HER2 receptors), both dependencies 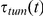 and 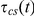 are very close (see part H in Figs. 4, 5 and 7).

It should be noted that intensity changes with time in the control experiments are similar in both tumor and the contralateral site, presenting exponential decay likely due to probe washout from the circulation with close characteristic times (part D in Figs. 4,5 and 7). For HER2 positive tumors, being imaged with HER2 specific probe, fluorescence intensity grows quickly during initial 2–3 hours after injection due to probe accumulation/binding to HER2 receptors and then decreases slowly due to probe unbinding (part D in Figs. 2 and 6).

The IHC results (Fig. 9) also indicate no expression of HER2 receptors (9A) and no accumulation of HER2-specific Affibody (9B) in MDA-MB-468 tumor. On the other hand, figures 9C-9F reveal high overexpression of HER2 in tumors of BT-474 and NCI-N87 types (3+ tumors according to conventional definition) and accumulation of HER2 Affibody at the surface of the cancer cells in these tumors. The quantitative values for HER2 expression in tumors, obtained by ELISA assays also confirm the IHC results.

Table 1 demonstrates statistical significance of the “binding” effect. We have used Wilcox Two-Sample U-test – Software R [40], to compare the tumor and contralateral site values of lifetime. The *p*-values were calculated based on the mean values of the measurement data at the tumor and contralateral site for three mice in each study. We combined the data in each subsample, because the lifetime data for three mice were very similar, as shown in Figs. 2, 4–7, part H. The *p*-value was calculated by comparing the measurements of the tumor and contralateral site in the range between 30 min to 5 hours after injection.

**Table 1:** Statistical significance of the measurements between 30 min to 5 hours at the tumor and contralateral site after injection, using Wilcox Two-Sample U-test.

	Tumor type				
	BT-474 with specific HER2 Affibody	BT-474 with non-specific HER2 Affibody	NCI-N87 with specific HER2 Affibody	NCI-N87 with non-specific HER2 Affibody	MDA-MB-468specific HER2 Affibody
**HER2 Specificity of the probe**: p-value to compare the fluorescence lifetime at the tumor and the contralateral sites	6.91E-05	0.7464	0.000588	0.8182	0.7491

The results show that in those cases, when HER2 binding can occur, there is a significant statistical difference between two time sequences of lifetime measurements, corresponding to the tumor and contralateral site (*p*-values<6e-4), while in the case of no probe binding the null hypothesis that both sequences are statistically similar holds (*p*-values>0.74). To our mind, these observations provide strong evidence that binding of the fluorescent ligands to receptors results in the noticeable reduction in the fluorescence lifetimes.

### Summary

In this study, we observed that the fluorescence lifetime of HER2 targeting optical probe changes after binding to HER2 receptors in the tumor area. The fluorescence lifetime is not sensitive to the intensity of excitation light and concentration of fluorophores, and this is one of the main advantages of fluorescence lifetime over fluorescence intensity measurements. The results show that comparison of the fluorescence intensity, originating from the tumor, with that of the contralateral site alone is not a good indicator of binding the fluorescent probe to the HER2 receptors. In the cases, when the Affibody did not bind to the HER2 receptors (e.g., Figs. 4B and 5B); there is an intensity contrast in the tumor are, relative to the contralateral site, probably due to the leakiness of tumor vascularization. To observe the binding of a fluorescent probe to a specific tumor receptor, determining the dynamic of the intensity contrast between tumor and contralateral site is required. This process involves imaging of the tumor for several hours after a probe injection. From practical point of view, the latter requirement may cause limitations for the clinical studies. On the other hand, the results of this study show that the difference between the fluorescence lifetime at the tumor and contralateral site is almost constant over time, and a relatively small time window would be sufficient to indicate the HER2 overexpression, making the technique more patient-friendly.

These results can help to create a new paradigm in the “image and treat” concept to assess the presence of specific cancer biomarkers and to monitor the influence of the drug on the tumor cells *in vivo*, especially in the early stage of the therapy. Application of time-resolved fluorescence imaging can reveal binding of specific optical probes to targeted tumor receptors *in vivo*. In addition to the clinical applications, this method can also be considered as a potential laboratory tool in pre-clinical animal studies and pharmaceutical research.
